# The Paths to Children’s Disordered Eating: The Implications of BMI, Weight-Related Victimization, Body Dissatisfaction and Parents’ Disordered Eating

**DOI:** 10.32872/cpe.v2i1.2689

**Published:** 2020-03-31

**Authors:** Marilou Côté, Maxime Legendre, Annie Aimé, Marie-Christine Brault, Jacinthe Dion, Catherine Bégin

**Affiliations:** a Laval University, Québec, Canada; b Université du Québec en Outaouais, St-Jérôme, Canada; c Université du Québec à Chicoutimi, Chicoutimi, Canada; Philipps-University of Marburg, Marburg, Germany

**Keywords:** weight-related victimization, disordered eating behaviors, body dissatisfaction, body mass index, children, cross-sectional study

## Abstract

**Background:**

Being the target of peer victimization is frequent among children categorized as overweight and obese and is thought to play a central role in disordered eating behavior development. In accordance with a previous theoretical model, this cross-sectional study aimed to replicate among children the mediating role of weight-related victimization from peers and body dissatisfaction in the association between body mass index (BMI) and children’s disordered eating attitudes and behaviors (CDEAB), while also taking into account the contribution of parents’ disordered eating attitudes and behaviors (PDEAB).

**Methods:**

Participants were 874 children aged between 8 and 12 years old who were recruited in elementary schools. Height and weight were measured and used to calculate BMI. Self-reported questionnaires were used to measure weight-related victimization, body dissatisfaction, CDEAB and PDEAB.

**Results:**

For both girls and boys, a path analysis showed no direct effect of BMI on CDEAB, but a significant indirect effect was found, indicating that weight-related victimization and body dissatisfaction mediated this relationship. In addition, the indirect effect of weight-related victimization and body dissatisfaction remained significant even when controlling for PDEAB.

**Conclusion:**

While weight itself appears to be insufficient to explain CDEAB, weight-related victimization may lead children to see their weight as problematic and develop disordered attitudes and behaviors toward eating. This suggests that weight-related victimization from peers and body dissatisfaction must be taken seriously and that preventive and intervention efforts must be pursued.

## Abstract (highlights)

- Body weight *per se* seems insufficient to explain children’s disordered eating attitudes and behaviors (CDEAB).
- Weight-related victimization and body dissatisfaction mediate the association between BMI and CDEAB.
- Parents‘ DEAB is associated with CDEAB.
- The tested paths from BMI to CDEAB appear to be globally the same for boys and girls.

Despite decades of efforts to prevent overweight and obesity, its prevalence is on the rise among children in developed and in developing countries (Ng et al., 2014). Children categorized as overweight or obese are at an elevated risk for disordered eating (Tanofsky-Kraff et al., 2004). Some public health programs designed to prevent overweight actually use weight stigmatization as a tool to sensitize people to the consequences of obesity (e.g., Georgia’s Strong4Life campaign; Teegardin, 2012). However, these programs may be counterproductive and instead increase weight-related victimization. In return, experiencing weight-related victimization may contribute to disordered eating among youth who present with overweight or obesity (Libbey, Story, Neumark-Sztainer, & Boutelle, 2008). Although there is existing literature linking weight-related victimization and eating behaviors, no research has examined this association while taking into account parents’ disordered eating, which has been extendedly related to children’s disordered eating (Scaglioni, Salvioni, & Galimberti, 2008). The current study mostly replicates previous work by assessing the mediating roles of weight-related victimization from peers and body dissatisfaction in the association between body mass index (BMI) and children’s disordered eating, and extends past reports by controlling for parents’ disordered eating.

Weight-related victimization includes cognitive and behavioral aspects. The cognitive aspect covers bias and stereotyping based on one’s weight. This leads to the belief that individuals categorized as overweight are lazy, lack self-discipline, have poor willpower, and show defects of intelligence and character. The behavioral aspect of weight-related victimization can materialize in verbal, physical and relational victimization, such as teasing, bullying, pushing and social exclusion (Puhl & Latner, 2007). Some studies demonstrated that children as young as 3 years old may be victimized because of their weight (Cramer & Steinwert, 1998; Rodgers, Wertheim, Damiano, Gregg, & Paxton, 2015). Therefore, weight-related victimization may start at a very young age. During the school years, weight-related victimization behaviors become frequent and mostly impact overweight children (see Puhl & Heuer, 2009; Puhl & Latner, 2007 for a review). For instance, many studies have shown that children and adolescents categorized as overweight are at a greater risk of being teased about their weight by school peers, educators, family members and peers of family members compared to their counterparts categorized as normal weight (Brixval, Rayce, Rasmussen, Holstein, & Due, 2012; Hayden-Wade et al., 2005; Neumark-Sztainer et al., 2002).

Among all weight-related victimization behaviors, teasing has been largely studied, most likely because it is common among youth (Hayden-Wade et al., 2005). Weight-related teasing is associated with various negative psychosocial consequences in children and adolescents, such as loneliness and preference for sedentary-isolative activities, social anxiety, poor quality of life and depression (Hayden-Wade et al., 2005; Juvonen, Lessard, Schacter, & Suchilt, 2017; Stevens, Herbozo, Morrell, Schaefer, & Thompson, 2017). Weight-related teasing also seems to be the starting point for many negative consequences related to eating and weight problems in adolescents. For example, parents, siblings and peer teasing were linked to body dissatisfaction in girls and to drive for muscularity in boys (Schaefer & Blodgett Salafia, 2014). Furthermore, weight-related teasing has been linked to the drive for thinness and disordered eating behaviors such as binge-eating, compensatory behaviors, and dietary restraint (Cook-Cottone et al., 2016; Haines, 2006; Neumark-Sztainer et al., 2002; Zuba & Warschburger, 2017). A recent longitudinal study noted that weight-related teasing in adolescence predicted resorting to disordered eating behaviors as a coping strategy, which in turn resulted in a higher body mass index (BMI) or into obesity 15 years later (Puhl et al., 2017).

Recently, the effect of weight-related teasing on disordered eating behaviors was validated in a few prospective studies. Most of these studies seemed to build their prospective design on a pioneering study by Thompson, Coovert, Richards, and Johnson (1995). Thompson and colleagues (1995) proposed a path analysis with a sample of girls aged 13-18 years old. In their model, the level of obesity at the baseline influenced weight-related teasing at the baseline, which further influenced body image (weight and appearance dissatisfaction) at the 3-year follow-up. Furthermore, body image at the 3-year follow-up influenced disordered eating behaviors such as bulimic behaviors and dietary restraint at the 3-year follow-up. Jendrzyca and Warschburger (2016) presented a similar comprehensive model of disordered eating behaviors in children. In their prospective design, 1,486 children aged 6-11 years old in Germany completed height and weight measurements (used for BMI calculation) and questionnaires related to eating, weight and body image (weight-related stigmatization, including weight-related teasing, body dissatisfaction and disordered eating behaviors) twice with a one-year interval. For girls, BMI at the baseline was significantly associated with the baseline weight-related stigma, which predicted body dissatisfaction one year later, which in turn predicted disordered eating behaviors, also at the one-year follow-up. For boys, a different pattern was found. BMI at the baseline was significantly associated with the baseline weight-related stigma, and body dissatisfaction at the one-year follow-up predicted disordered eating behaviors at the one-year follow-up, but baseline weight-related stigma did not predict body dissatisfaction at the one-year follow-up. Using a similar model, Pryor and colleagues (2016) found that children categorized as overweight and targeted by peers’ victimization between 6 and 12 years old tended to be less satisfied with their bodies (they wanted to be thinner) and to report increased depression and anxiety at 13 years old.

Thereby, some authors implied that weight-related victimization should be included in a comprehensive model of disordered eating behaviors development (Jendrzyca & Warschburger, 2016). However, most available studies have only targeted adolescent populations. Furthermore, studies tend to report mixed results regarding possible sex specific effects, and parental influences are often overviewed. However, parents’ eating behaviors have a major influence on their children’s eating behaviors, especially at a younger age (Scaglioni, et al., 2008; Ventura & Birch, 2008; Wertheim, Martin, Prior, Sanson, & Smart, 2002; Wertheim, Mee, & Paxton, 1999). Therefore, to better assess (and not overestimate) the influence of weight-related victimization and body dissatisfaction in a comprehensive model of disordered eating behaviors in children categorized as overweight or obese, the influence of parents’ eating behaviors should be considered.

The present study aimed to examine the mediating role of 1) weight-related victimization from peers, as perceived by children, and 2) body dissatisfaction in the association between BMI and children’s disordered eating attitudes and behaviors (CDEAB) among 8-12 years old boys and girls, controlling for parents’ disordered eating attitudes and behaviors (PDEAB). It was expected that a higher BMI would be associated with greater CDEAB, mediated by perceived weight-related victimization and body dissatisfaction (serial) for both boys and girls. Moreover, it was hypothesized that PDEAB would be positively associated with CDEAB.

## Method

### Participants

Participants were 874 children aged between 8 and 12 years old and one of their parents. They were recruited from 27 public elementary schools located in two urban areas in the province of Quebec, Canada. The sample was composed of 44% boys and 56% girls. Their mean age was 10.29 (*SD* = 1.19). Among the sample, 1.5% of the children could be classified in the underweight category, 69.3% in the normal weight category, 20.9% in the overweight category and 8.3% in the obese category. Regarding weight-based victimization, 24.4% of children reported having been teased about their weight at least once. The participating parents were mostly mothers (86%). Their mean age was 39.65 years old (*SD* = 5.69), and their mean BMI was 26.23 (*SD* = 5.04). Almost all of the children were born in Canada (95%) and came from a family where their parents were either married or living in a common-law relationship (83%). On average, these children came from wealthy and educated families. Nearly a third had an annual family income of $100,000 or more, which was over the average wage (approximately $73,000) in the province of Quebec (Statistics Canada, 2019). Furthermore, almost the half of the children had a parent with a university diploma, while about 35% of the population of the province of Quebec had achieved an academic degree (Crespo, 2018).

### Procedure

The children were recruited to participate in a study about body weight, body image and eating and physical activity habits. The study was presented to them in class. Interested children were given an envelope containing both parents and children questionnaires, as well as informed consent form. Both the children and parents were asked to complete questionnaires at home (approximately 45 minutes for parents and 30 minutes for children). Parents were instructed to let their children fill autonomously the questionnaires. Children returned the completed questionnaires to their teacher, and were met individually at school by a trained research assistant to collect their anthropometric (height and weight) measures. All of the parents gave written informed consent (approved by University’s Institutional Review Board of Laval University) prior to their inclusion in the study, and children provided their assent to participate. The children who completed the questionnaires were included in a lottery drawing to win a $100 gift card to a sports shop.

### Measures

#### Children’s BMI

Height and weight were measured individually and out of sight of the children’s peers and only one time as recommended by Lohman, Roche, and Martorell (1988), and trained research assistants used a metric scale and a numeric weighing scale. Height was measured to the nearest 0.1 centimeter and weighed to the nearest 0.2 pound. Measurements in pounds were then transformed into kilograms. Gender specific BMI-for-age *z* scores were computed based on the World Health Organization recommendations (WHO Multicentre Growth Reference Study Group, 2006). The children’s BMI was classified into four categories (underweight, normal weight, overweight, or obese) still according to the WHO recommendations. These categories were used to describe the sample and for the mean comparisons, and BMI *z*-scores were used as a continuous variable in the path analyses.

#### Perceived Weight-Related Victimization by Peers

Perceived weight-related victimization was measured with a question adapted from the Children's Social Experience Questionnaire (Crick & Grotpeter, 1996). The question “How often does another child say negative things about your weight?” was answered on a 5-point Likert scale ranging from 1 (never) to 5 (all the time). A higher score indicated a higher level of perceived weight-related victimization by peers.

#### Body Dissatisfaction

Body dissatisfaction was evaluated with two questions inspired by Collins (1991). One evaluated actual body perception (How would you describe your body? With answers ranging from 1 “far too thin” to 5 “far too big”), while the other evaluated desired body (How would you like your body to be? With answers ranging from 1 “a lot thinner” to 5 “a lot bigger”). We further subtracted the desired body from the actual body perception. The discrepancy between the perceived and the desired body provided an indication of the level of body dissatisfaction, with a negative score reflecting a desire for a thinner body and a positive score reflecting a desire for a larger body.

#### Children’s Eating Attitudes Test

The children’s version of the Eating Attitudes Test (ChEAT; Maloney, McGuire, & Daniels, 1988) was used to measure disordered eating attitudes and behaviors. The ChEAT is a 26-item self-report questionnaire, with a 6-point Likert scale ranging from 1 (never) to 6 (always). The total score was used. A higher score reflects more disordered eating attitudes and behaviors. Its reliability and concurrent validity have been demonstrated previously (Maloney, McGuire, Daniels, & Specker, 1989; Smolak & Levine, 1994). The Cronbach’s alpha was .79 in the present sample.

#### Eating Attitudes Test

The Eating Attitudes Test (EAT-26; Garner, Olmstead, Bohr, & Garfinkel, 1982) was used to measure parents’ disordered eating attitudes and behaviors. The EAT is a 26-item self-report questionnaire which uses a 6-point Likert scale ranging from 1 (never) to 6 (always). The total score was used. A higher score reflects more disordered eating attitudes and behaviors. The questionnaire has adequate reliability (Koslowsky et al., 1992). The Cronbach’s alpha was .87 in the present study.

#### Statistical Analyses

Prior to analyses, all variables’ distributions were inspected, and appropriate transformations were applied when needed in order to respect the basic assumptions. First, *t*-test and ANOVA analyses were run to compare the children on the three study dependent variables (weight victimization, body dissatisfaction and CDEAB) based on their sex and BMI category. Afterward, the proposed model was tested with a path analysis using Mplus version 7.0 (Muthén & Muthén, 2012). Path analysis is a statistical method that allows the simultaneous testing of both direct and indirect associations among different variables (Kline, 2011).

In this model, BMI was used as an independent variable with both weight-related victimization and body dissatisfaction as mediators (serial mediation), and CDEAB was used as the dependent variable. PDEAB was included as a control variable. Because standard errors underlying indirect effects (i.e., product terms) are known to be skewed, we instructed Mplus to generate 1000 bootstrap samples from the data to create indirect effects with bias-corrected 95% confidence intervals (CIs; MacKinnon, Lockwood, & Williams, 2004). Indirect effects would only be found to be significant if the CIs would not include zero.

To determine whether the model provided a good fit for the data, three indices recommended by Hu and Bentler (1999) were used: the Comparative Fit Index (CFI), the standardized root mean square residual (SRMR), and the root mean square error of approximation (RMSEA). The determined threshold values indicating a good fit are CFI ≥ .95, SRMR ≤ .08, and RMSEA ≤ .06 (Hu & Bentler, 1999). A good fit of the model can also be identified by a nonsignificant χ2 value (Tabachnick & Fidell, 2001).

## Results

### Mean Comparisons

The results from *t*-tests and ANOVAs, as well as means and standard deviations, are presented in Table 1. Weight-related victimization was similar for boys and girls but significantly differed across weight statuses. Children categorized as obese reported more frequent weight-related victimization compared to children categorized as underweight, normal weight and overweight (all *p* values < .001). Children categorized as overweight also reported more victimization than peers categorized as normal weight (*p* < .001). Body dissatisfaction differed between boys and girls, as well as across weight statuses. As expected, girls were significantly more dissatisfied with their body than boys. Children categorized as obese were more dissatisfied with their body than children categorized as underweight, normal weight and overweight (all *p* values < .01). Children categorized as overweight were also more dissatisfied than children categorized as normal weight (*p* < .001). Finally, for CDEAB, girls reported significantly higher scores than boys. Across weight statuses, children categorized as obese reported more disordered eating attitudes and behaviors than children categorized as overweight or normal weight (all *p* values < .01).

**Table 1:** Means and Standard Deviations by Sexes and by Weight Categories

Variable	Sex				Weight category								*t*	*F*
	Girls		Boys		Underweight		Normal		Overweight		Obesity		Sex	Weight category
	*M*	*SD*	*M*	*SD*	*M*	*SD*	*M*	*SD*	*M*	*SD*	*M*	*SD*		
Weight-related victimization	1.40	0.80	1.36	0.74	1.23	0.60	1.24	0.58	1.53	0.88	2.17	1.19	-0.72	39.50***
Body dissatisfaction	0.62	0.95	0.49	0.84	0.75	1.29	0.36	0.74	0.80	0.94	1.64	1.05	-2.17*	59.04***
CDEAB	6.44	5.56	5.55	3.98	5.62	4.31	5.52	4.19	6.49	5.50	9.40	7.59	-1.60*	10.20***

*Note.* *N* = 874 children. CDEAB = children’s disordered eating attitudes and behaviors.

*p < .05. ***p < .001.

### Path Analyses

Pearson’s correlations between the variables studied are presented in Table 2. The proposed theoretical model was first tested with path analyses separately for both boys and girls. The results showed very similar patterns among boys and girls. Therefore, we expected the models to be invariant with regard to sex and we performed multigroup tests. The nonsignificant adjusted difference of the chi-square, χ^2^(5) = 6.579, *p* = .254, showed that the model was invariant by sex on all the tested paths except the BMI-body dissatisfaction one. That is, the tested paths were similar for boys and girls, but the path between BMI and body dissatisfaction was slightly different regarding the strength of the association, β = .33 (*p <* .0001) for girls and β = .18 (*p* = .003) for boys. Since this minor sex difference did not affect the direction nor the signification of the association between BMI and body dissatisfaction, a single model will be presented for girls and boys for the sake of parsimony.

**Table 2:** Pearson’s Correlations Between Studied Variables

Variable	1	2	3	4	5
1. CDEAB	–	.09**	.16**	.22**	.29**
2. PDEAB		–	.08*	.04	.05
3. BMI			–	.21**	.32**
4. Weight-related victimization				–	.31**
5. Body dissatisfaction					–

*Note.* *N* = 874 children. CDEAB = children’s disordered eating attitudes and behaviors. PDEAB = parents’ disordered eating attitudes and behaviors.

*p < .05. **p < .01.

**Figure 1:**
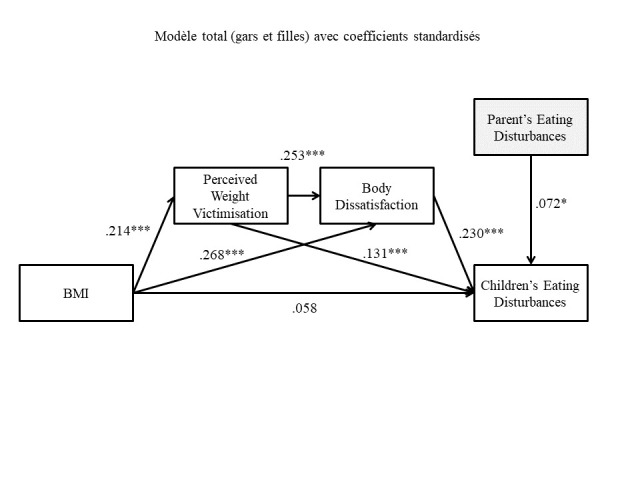
Relationships Among Studied Variables in Boys and Girls, With Standardized Coefficients *Note.* *N* = 874 children. *p < .05. **p < .01. ***p < .001.

The fit indices revealed that the tested model provided a good fit to the data: CFI = .99, SRMR = .02, RMSEA = .03. The nonsignificant chi-square value also indicated that the data were adequately represented by the model, χ^2^(3) = 5.59, *p* = .133. The model with standardized path coefficients is presented in Figure 1. The model explained 11% of the variance of the main dependent variable (CDEAB; *R*^2^ = .11). 

In this model, BMI did not have a direct effect on CDEAB (β = .06; *p* = .097). Rather, three different paths (indirect effects) were statistically significant: 1) BMI was associated to CDEAB through weight-related victimization and body dissatisfaction (β = .01, 95% bootstrap CI [.001, .005]; 2) BMI was associated to CDEAB through perceived weight-related victimization (β = .03, 95% bootstrap CI [.003, .012]; and 3) BMI was associated to CDEAB through body dissatisfaction (β = .06, 95% bootstrap CI [.010, .021]. The results of the path analyses further confirmed the relevance of adding the control variable PDEAB, since its positive association with CDEAB was significant (β = .07; *p* < .05).

## Discussion

The aim of this study was to mostly replicate previous work (Jendrzyca & Warschburger, 2016; Thompson et al., 1995) by examining the mediating role of weight-related victimization from peers as perceived by children aged 8 to 12 years old and body dissatisfaction in the association between BMI and CDEAB, and to extend previous studies by taking into account the contribution of PDEAB. Overall, the results confirmed our hypotheses and revealed that BMI was associated with disordered eating only through its associations with perceived weight-related victimization and body dissatisfaction. Parental disordered eating was also associated with higher disordered eating among children.

First, the level of perceived weight-related victimization and body dissatisfaction were significantly different across weight statuses. Children categorized as overweight or obese reported more weight-related victimization and body dissatisfaction compared to children categorized as normal weight. This is consistent with what others have previously reported (Brennan, Lalonde, & Bain, 2010; Neumark-Sztainer et al., 2002; Puhl & Latner, 2007). The level of perceived weight-related victimization was similar for boys and girls, but girls were significantly more dissatisfied with their body than boys were. This may be because girls, even at this age, present a higher risk of being exposed to media and beauty pressure, resulting in higher preoccupation with their weight and body shape. It could also be that for boys, body dissatisfaction kicks in later or that it may be more about looking fit and muscular than looking thin (Barlett, Vowels, & Saucier, 2008; Brennan et al., 2010; Dion et al., 2016; Thompson & Chad, 2000).

Even though girls reported more body dissatisfaction than boys did, the same trajectory from BMI to CDEAB applied for both sexes, since the model was, globally, statistically invariant in regard to sex. Considering that BMI had no direct effect on CDEAB, weight *per se* appears to be insufficient to explain the development of disordered eating attitudes and behaviors. Most likely, it is the negative experience, mostly interpersonal, associated with being categorized as overweight or obese that may influence children and adolescents to see their weight as problematic. As demonstrated in this study, high BMI was associated with CDEAB through the indirect effect of perceived weight-related victimization and body dissatisfaction. Furthermore, BMI was also associated with CDEAB through the indirect effect of perceived weight-related victimization and body dissatisfaction separately. Along with the findings of Jendrzyca and Warschburger (2016), the present results suggest that weight-related victimization and body dissatisfaction might play a key role in the likelihood of developing disordered eating attitudes and behaviors for children who present as overweight or obesity. This highlights the need to fit in, as children grow older, and the important effect that these relationships with peers have on children. In addition, it may provide a clue about why body dissatisfaction is different between girls and boys. This might be likely because the importance of interpersonal experiences may change greatly from childhood to adolescence and differently for girls and boys. However, the cross-sectional design of the present study calls for caution, and additional prospective studies are needed to confirm those hypotheses.

The fact that our study took into account the contribution of PDEAB was an important strength. While the association between PDEAB on CDEAB does not need to be proven further (Scaglioni et al., 2008; Ventura & Birch, 2008; Wertheim et al., 2002; Wertheim et al., 1999), it still has to be considered when predicting CDAEB in order to avoid overestimating the effect that weight-related victimization has on it. Had we not statistically controlled for PDEAB, one could have thought that the association between BMI, weight-related victimization, body dissatisfaction and CDAEB may be explained by parental influences. However, although parents may influence the development of disordered eating in their children as they approach adolescence, these youths may be even more affected by their experiences with peers. As they get older, negative experiences such as weight-related victimization can seriously affect the way children evaluate themselves and push them to try to modify their weight and appearance to like themselves better and better fit in their peer group (Vander Wal, 2012). Another strength of this study was to target elementary school girls and boys. Studies that focus on weight-related victimization and body dissatisfaction have previously targeted, for the most part, high school adolescents. It appeared important to replicate the results from adolescents’ studies with younger children since disordered eating attitudes and behaviors can be adopted early and can be especially harmful (Goldschmidt, Aspen, Sinton, et al., 2008). The recruiting process is another important element of this study. To favor a diversified sample, 874 children from 27 public elementary schools were included in our path analysis. Finally, it was a great strength to use objective anthropometric measures because parents are likely to misreport their children's weight and height (Brault, Turcotte, Aimé, Côté, & Bégin, 2015).

Some limitations of this study should be considered. First, as mentioned earlier, the cross-sectional design does not allow for drawing causal conclusions. However, the paths proposed follow a logical cascade in time that has already been demonstrated in a prospective design (Jendrzyca & Warschburger, 2016). Second, weight-related victimization from peers and body dissatisfaction were measured with single items. Moreover, no specific time frame was given in the question assessing victimization. The use of validated questionnaires for our two mediating variables would have significantly enhanced internal validity. Since the same measurement limitation applies to the prospective study of Jendrzyca and Warschburger (2016), future studies may benefit from testing weight-based victimization and body dissatisfaction with complete validated scales. Nonetheless, despite the limitation that represents the use of single item measures (i.e., underestimation of the strength of the tested associations; Menzel et al., 2010), the present study successfully detected statistically significant effects between studied variables, which suggests robust associations. Another limitation stems from the representativeness of the sample. Indeed, higher-educated wealthy families were over represented. Since disordered eating behaviors and body dissatisfaction have been previously found to be higher in high socioeconomic status (SES) children compared to low SES children (Adams et al., 2000; O’Dea & Caputi, 2001), it would be of great interest to replicate our results in a more diversified sample in terms of SES. Additionally, it would be of great interest to assess victimization from different points of view, (i.e., reported not only from children but also from teachers and parents) to verify whether it is weight victimization *per se* which is associated with negative psychological outcomes or feeling victimized. Different sources of comments should also be studied, since parental comments on weight might be very harmful for young people (Neumark-Sztainer et al., 2010).

### Conclusion

This study adds to the limited data currently available in the field of the early development of disordered eating behaviors (before adolescence). An important contribution of this study was to consider the implication of PDEAB in a comprehensive model of eating attitudes and behaviors in children. A model in which weight-related victimization experienced by children was associated with body dissatisfaction and disordered eating attitudes and behaviors was replicated. While weight itself appears to be insufficient to explain disordered eating, interpersonal experiences might be what influence children to see their weight as problematic and adopt disordered attitudes and behaviors toward eating. This suggests that weight-related victimization from peers and body dissatisfaction must be taken seriously and that prevention and intervention efforts must be pursued.
